# A Bioinspired Peptide in TIR Protein as Recognition Molecule on Electrochemical Biosensors for the Detection of *E. coli* O157:H7 in an Aqueous Matrix

**DOI:** 10.3390/molecules26092559

**Published:** 2021-04-28

**Authors:** Jose Luis Ropero-Vega, Joshua Felipe Redondo-Ortega, Yuli Juliana Galvis-Curubo, Paola Rondón-Villarreal, Johanna Marcela Flórez-Castillo

**Affiliations:** 1 Facultad de Ciencias Exactas, Naturales y Agropecuarias, Ciencias Básicas y Aplicadas Para la Sostenibilidad—CIBAS, Universidad de Santander, Calle 70 No. 55-210, Bucaramanga C.P. 680003, Santander, Colombia; julianagalvis20@outlook.com (Y.J.G.-C.); johanna.florez@udes.edu.co (J.M.F.-C.); 2 Facultad de Ciencias de la Salud, Grupo de Investigación en Biología Molecular y Biotecnología, Universidad de Santander, Calle 70 No. 55-210, Bucaramanga C.P. 680003, Santander, Colombia; diseno.molecular@udes.edu.co

**Keywords:** pathogen, PEPTIR-1.0, screen-printed electrodes, gold nanoparticles, electrodeposition, limit of detection, selectivity

## Abstract

Currently, the detection of pathogens such as *Escherichia coli* through instrumental alternatives with fast response and excellent sensitivity and selectivity are being studied. Biosensors are systems consisting of nanomaterials and biomolecules that exhibit remarkable properties such as simplicity, portable, affordable, user‑friendly, and deliverable to end‑users. For this, in this work we report for the first time, to our knowledge, the bioinformatic design of a new peptide based on TIR protein, a receptor of Intimin membrane protein which is characteristic of *E. coli*. This peptide (named PEPTIR‑1.0) was used as recognition element in a biosensor based on AuNPs‑modified screen‑printed electrodes for the detection of *E. coli*. The morphological and electrochemical characteristics of the biosensor obtained were studied. Results show that the biosensor can detect the bacteria with limits of detection and quantification of 2 and 6 CFU/mL, respectively. Moreover, the selectivity of the system is statistically significant towards the detection of the pathogen in the presence of other microorganisms such as *P. aeruginosa* and *S. aureus*. This makes this new PEPTIR‑1.0 based biosensor can be used in the rapid, sensitive, and selective detection of *E. coli* in aqueous matrices.

## 1. Introduction

The World Health Organization (WHO) highlights the need for constant monitoring and control of pathogenic microorganisms such as *Escherichia coli*, because strains such as the Shiga toxin‑producing pathotype cause severe diseases in humans after ingestion through contaminated food or water. This represents a tremendous public health problem [1,2]. Several methods have been developed to detect *E. coli* O157:H7 in aqueous matrices, including traditional culture with selective media [3], serotyping with specific antibodies against O157 and H7 antigens [4,5], the amplification of specific genes by PCR [6,7], or hybridization of virulence genes by DNA microarrays [8]. However, these methods are limited by high cost, use of specialized equipment, long sample processing time and the need for personnel trained in sample handling and analysis. For this reason, it is necessary to design devices that are affordable, sensitive, specific, user-friendly, rapid and robust, equipment-free, and deliverable to end-users in order to facilitate the monitoring of the microbiological quality of aqueous systems [9].

Biosensors are instruments consist of a molecule of biological nature, also called biological recognition element, and a physicochemical transducer [10]. The recognition element is responsible for interacting biochemically with the analyte of interest and gives the specificity to the biosensor. In principle, any biological structure with this capacity can be used. Whole cells, subcellular organelles, fine sections of cellular tissues, membrane receptors, nucleic acids (DNA or RNA), antibodies, enzymes, and peptides are commonly used [11,12,13]. On the other hand, the transducer is responsible for transform the biochemical interaction between the recognition element and the analyte into a quantifiable signal [14,15,16]. In electrochemical biosensors, this quantifiable signal commonly consists of a current or changes in the impedance of the biosensor/analyte interface. On this devices, transducers are commonly based on conductive materials like carbon nanostructures and metallic nanoparticles [17]. In this sense, biosensors become an interesting alternative method for the detection of microorganisms such as *E. coli* in aqueous matrices.

Recently, peptide-based biosensors have been widely studied in pathogen detection and disease diagnosis due to their sensitivity, low cost, and specificity [18,19,20,21,22]. These devices are probably the most versatile systems due to the physicochemical properties of the peptides. These molecules are typically composed of 10 to 100 amino acids and adopt secondary structures such as α‑helix, β‑sheet and loops. These tridimensional structures can interact with analytes of interest such as ions, molecules, membranes of microorganisms or whole cells. The peptide‑analyte interaction occurs commonly through non-covalent intermolecular interactions such as hydrophobic interactions, hydrogen bonds, electrostatic interactions, etc. [23]. Furthermore, peptides can be designed in such a way that they can replace the domains of interest of some proteins, such as specific regions of antibodies, enzymes, or protein receptor binding sites [24]. Thereby, it is possible to obtain shorter molecules with higher stability and specificity.

The aim of this study was to design and evaluate a peptide analogous to the TIR protein (Translocated Intimin Receptor) as recognition molecule in electrochemical biosensors for the detection of *E. coli* in aqueous matrices. The TIR protein is a molecule expressed by the TIR gene of *E. coli* and is involved in the pathogenesis mechanism of enterohemorrhagic and enteropathogenic serotypes towards the outer membrane of enterocytes. TIR interacts through the extracellular domain of *E. coli* with the protein Intimin which is an outer membrane adhesin encoded by the eaeA gene, widely studied due to its role in the pathogenesis of *E. coli* [25]. This protein was considered in this study as a target receptor for the detection of this pathogen.

It is important to highlight that this is the first study in which bioinformatics tools and electrochemical techniques are integrated to design peptides analogous to the TIR protein and used as recognition molecules for the electrochemical detection of *E. coli*.

## 2. Results and Discussion

### 2.1. Selection of the TIR Protein Interaction Sequence Using Bioinformatics Tools

**Figure 1:**
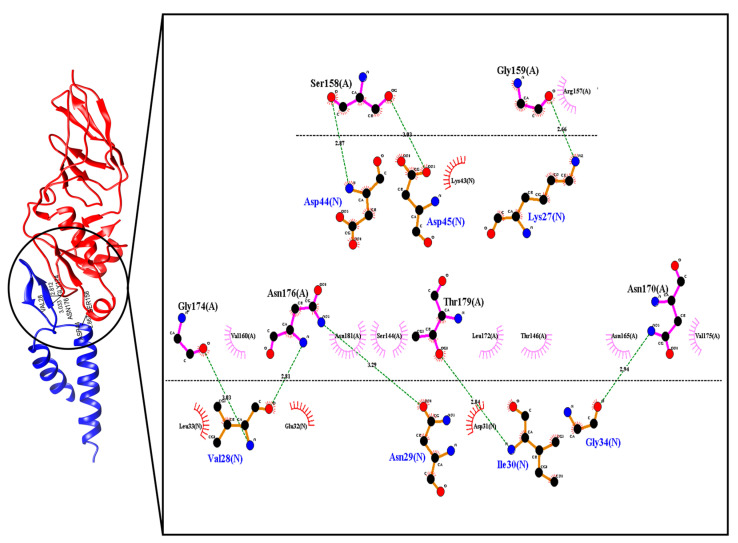
Crystal structure of the interaction between TIR proteins (N-Blue chain) and Intimin (A-Red chain), obtained from the PDB 2ZQK model.

Figure 1 shows the domains of possible interaction between the TIR and Intimin proteins. TIR protein binding domain has 20 amino acids responsible for interaction with the Intimin protein. Within these amino acids are four acidic amino acids (one Glu residue and three Asp residues) and two Lys residues, which confers a net negative charge on the binding domain. Additionally, seven hydrophobic residues are observed, suggesting that hydrophobic and electrostatic interactions are predominant in the TIR‑Intimin interaction [26].

**Figure 2:**
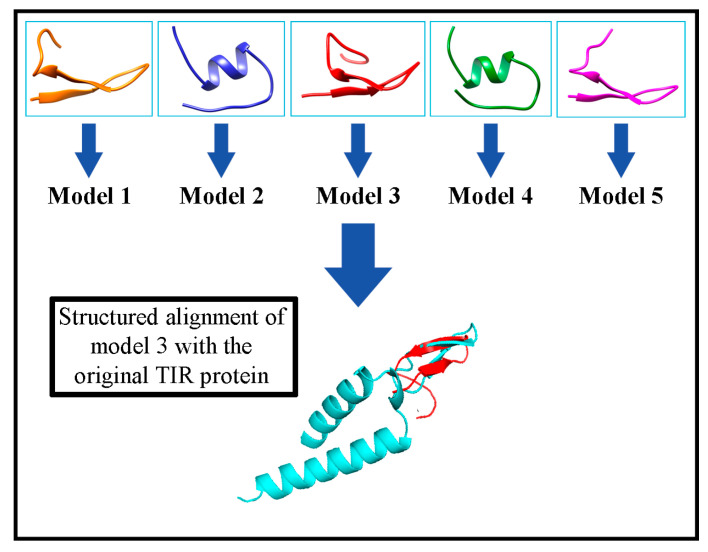
Three-dimensional structure of five models obtained from the sequence: QKVNIDELGNAIPSGVLKDD with the PEP-FOLD tool.

The 20 amino acids of the binding domain between TIR-Intimin (QKVNIDELGNAIPSGVLKDD) were used to design the peptide to be immobilized in the electrochemical biosensor. 3D‑structure of peptide was obtained using PEP-FOLD program. Figure 2 shows the five most probable models which were compared with the binding domain of the TIR protein of the 2ZQK model using the PyMol program. Models 1, 3 and 5 present a conserved structure where it can be seen two β-sheets similar to those seen in the TIR protein. Otherwise, models 2 and 4 present an α‑helix structure different from the TIR protein structure.

Root-mean-square-deviation of atomic positions (RMSD) values were determined to quantify the structural differences between the models found by PEP-FOLD and the binding domain of the TIR protein (Table 1).

Models 2 and 4 show the highest RMSD values due to the α‑helix structure acquired in the IDELGNA amino acid region. Model 5 acquired a disordered structure at the C-terminal, which is distant from the TIR protein loop. For this reason, it shows a high RMSD value despite having a similar structure to the TIR protein. Model 3 presented the lowest RMSD value and was therefore selected to perform the molecular docking simulations.

### 2.2. Selection of Ligands and Molecular Docking between the Peptide and the Intimin Protein

**Figure 3:**
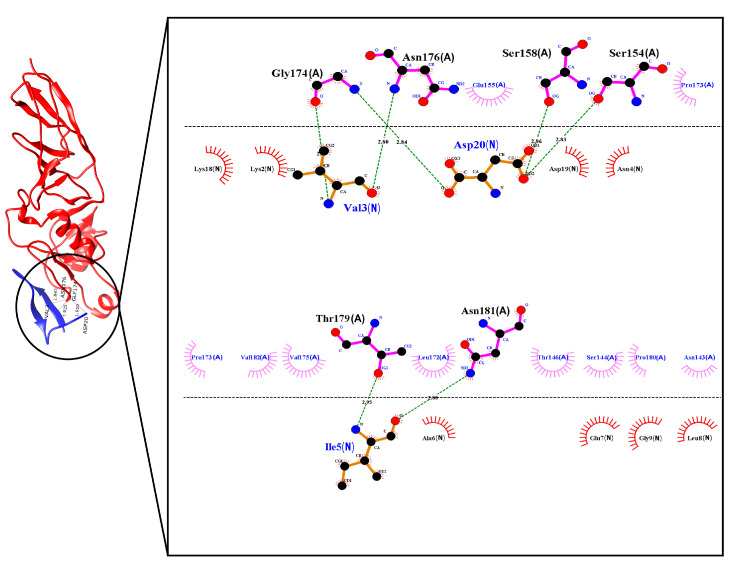
Complex between the peptide model obtained with PEP-FOLD and the original structure of the Intimin protein, predicted by the FlexPepDock program.

The selected recognition molecule was named as *N*‑chain to perform a molecular docking analysis together with the original structure of the Intimin protein (Figure 3), identified as chain A. Using the FlexPepDock platform, the site of interaction of the peptide based on the TIR protein in the extracellular domain of the Intimin protein was confirmed.

From the 200 models calculated by the FlexPepDock program, the five models with the best Rosetta and RMSD energy score values were further analyzed using the software LigPlot+ [27], the Prodigy Haddock program [28] and the Rosetta InterfaceAnalyzer protocol [29]. The results obtained are shown in Table 2. The number and type of intermolecular contacts were defined within the distance limit of 5.5 Å. dG_Sep gives the binding energy calculated as the change in Rosetta energy when the interface forming chains are separated versus when they are complexed (Rosetta energy units). dSASA indicates the solvent accessible area buried at the interface. Per_res_energy is the average energy of each residue at the interface (Rosetta energy units).

The interactions predicted by LigPlot+ showed that the designed peptide and TIR protein had the same binding region when interacting with the Intimin protein. Moreover, even though the interaction occurs with a ligand of only 20 amino acids, it manages to generate more than 80% of the number of intermolecular contacts within the 5.5 Å distance limit that can occur at the peptide and Intimin interface, with a total of 52 interfacial contacts. Furthermore, model 3.1 generated by the FlexPepDock program presents values of binding affinity and dissociation constant close to the values of the original model, as shown in Table 2. Moreover, for the parameters obtained with the InterfaceAnalyzer protocol, i.e., the binding energy, the solvent accessible area buried at the interface, and the average energy of each residue at the interface, the values obtained for the five best models of peptide-Intimin complexes were better than the values obtained for the TIR-Intimin complex.

These results are promising, and it can be inferred that, although the peptide model is much smaller than the original ligand, the interactions that define the binding with the receptor molecule are mainly concentrated in its 20 residues and predict the ability of the peptide to interact appropriately in vitro with the protein Intimin. Therefore, this peptide was selected as a recognition molecule (designated as PEPTIR‑1.0) for the subsequent preparation of the electrochemical biosensor.

### 2.3. Preparation and Characterization of the Biosensor

#### 2.3.1. Electrodeposition of Gold Nanoparticles (AuNPs)

**Figure 4:**
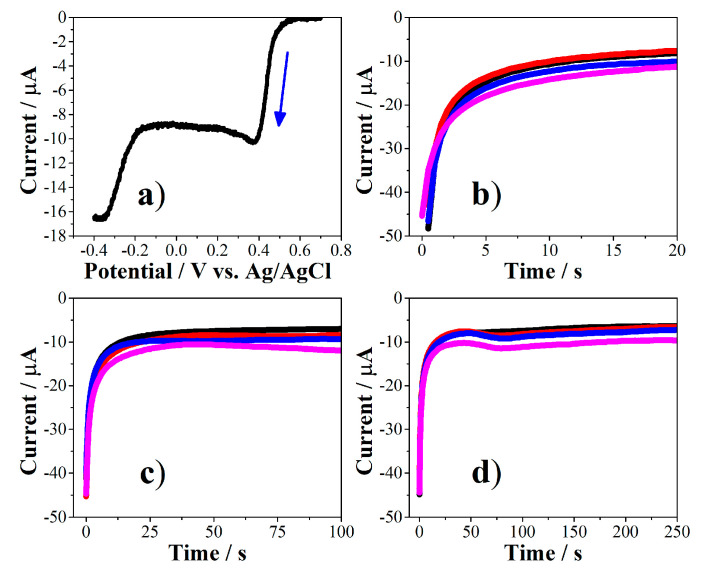
Electrodeposition of gold nanoparticles (AuNPs) on screen‑printed electrodes. Linear sweep voltammogram (5.0 mV/s) of gold precursor (**a**). Current vs. time curves at 20 s (**b**), 100 s (**c**) and 250 s (**d**). The applied potentials were +0.05 V (black lines), −0.05 V (red lines), −0.15 V (blue lines) and −0.25 V (rose lines) in all cases.

The AuNPs were deposited on the working electrode (WE) of the screen‑printed electrodes (SPE, Italsens) using the chronoamperometry (CA) technique. An aqueous solution of 1.0 mM of HAuCl_4_ × H_2_O in 0.5 M of H_2_SO_4_ was used as gold precursor. The potentials selected for CA were based on the result obtained by linear sweep voltammetry. The effect of potential and time used in CA was evaluated on the preliminary detection of *E. coli* (EC, 500 CFU/mL). The results of the analyses are shown in Figure 4.

Linear sweep voltammogram (Figure 4a) shows an onset reduction potential to a close value of +0.6 V with a maximum current at +0.4 V which is maintained until −0.2 V. Below this potential, the reduction of the medium to produce hydrogen gas begins [30]. Given the above, the potentials selected for the electrodeposition of gold by CA were below +0.05 V to ensure the reduction of the gold precursor. All the CA curves (Figure 4b–d) exhibited the typical shape and current values for an electrochemical reduction process [31]. It is noteworthy that the reduction currents reach a steady state value close to −10 μA after about 20 s in all cases. The currents were slightly more negative when the most negative reduction potentials (−0.15 and −0.25 V, blue and rose lines, respectively) were applied. On the other hand, it was not observed a significant difference in the currents obtained when applying the potentials of +0.05 and −0.05 V at the different electrodeposition times.

**Figure 5:**
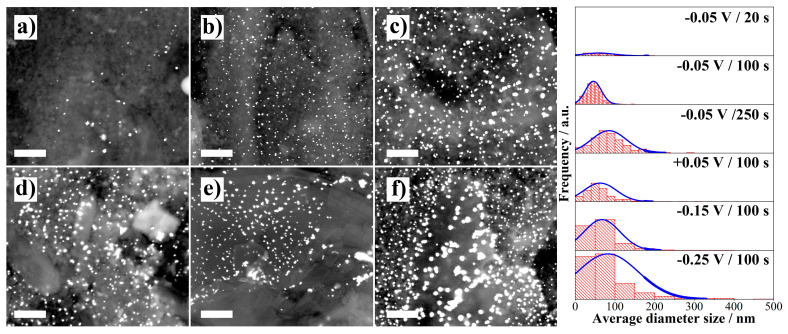
Scanning electron microscopy (SEM) results. Left: Micrographs of the working electrodes with AuNPs prepared by electrodeposition at a constant potential of −0.05 V during (**a**) 20 s, (**b**) 100 s and (**c**) 250 s, and at a constant time of 100 s at (**d**) +0.05 V, (**e**) −0.15 V and (**f**) −0.25 V. All images were recorded with backscattered electron detector (BSED). 50,000 X, scale bar 1 μm. Right: Histograms of the average diameter size of AuNPs for each condition.

The surface of the WE was analyzed by scanning electron microscopy to evaluate the size and distribution of the gold nanoparticles formed. The results are shown in Figure 5.

The effect of increasing the electrodeposition time at a constant potential of −0.05 V (Figure 5a–c) showed the formation of a greater number of particles. As expected according to the CA results, the nucleation stage occurs during first 20 s followed by the growth stage, reaching average particle sizes of around 90 nm at 250 s. In the nucleation stage, few particles are formed while there is a good distribution and size of them in a time of 100 s (see histograms in right of Figure 5). On the other hand, it was observed a large size distribution when different potentials were applied at a constant time of 100 s (Figure 5d–f and histograms). It is worth noting that at electrodeposition potentials of −0.15 and −0.25 V, many particles are observed but with a very varied size distribution.

**Figure 6:**
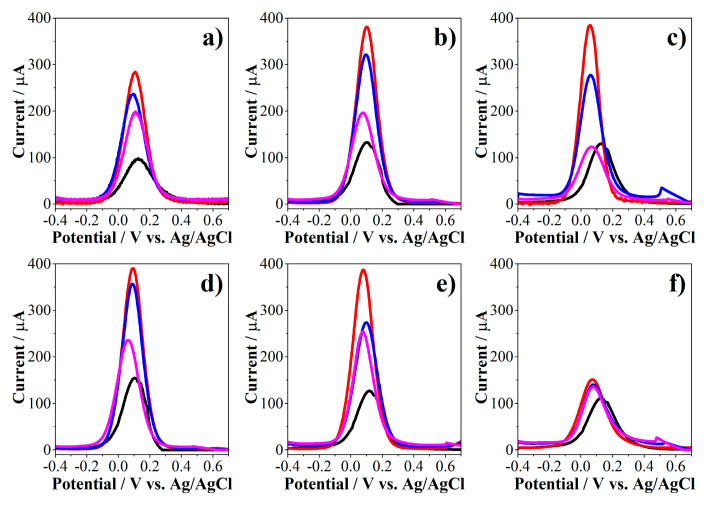
SWV results of the effect of varying the electrodeposition time for (**a**) 20 s, (**b**) 100 s and (**c**) 250 s at a constant potential of −0.05 V and varying the applied potential at (**d**) +0.05 V, (**e**) −0.15 V and (**f**) −0.25 V at constant time of 100 s in the electrodeposition of AuNPs towards the detection of *E. coli* (500 CFU/mL). The curves correspond to SPE (black lines), SPE/AuNPs (red lines), SPE/AuNPs/PEP (blue lines) and SPE/AuNPs/PEP/EC (rose lines) in all cases. 10 mM of [Fe(CN)_6_]^−3/−4^ in 0.1 M of KCl aqueous solution.

The definition of the electrodeposition variables of AuNPs by CA was carried out with the implementation of a factorial design of two factors in the detection of *E. coli* (500 CFU/mL, concentration used as reference). This design allows to study the simultaneous effect of the reduction applied potential and the electrodeposition time and how these factors influence the response of the entire biosensor construction and the detection of *E. coli*. Square wave voltammetry (SWV) was used to evaluate the changes in the electrochemical response of the biosensor in the presence of [Fe(CN)_6_]^−3/−4^ couple as redox indicator. The PEPTIR‑1.0 was used as recognition element and 10 μL of an aqueous solution of 500 nM were used for its immobilization on the WE. The results of the effect of the electrodeposition potential at a constant time of 100 s and varying the electrodeposition time at a constant potential of −0.05 V is shown in Figure 6. The results of the other potentials and times are found in supplementary information (Figures S1 and S2).

The voltammograms in Figure 6 show an increase in the current of the AuNPs‑modified screen‑printed electrodes (SPE/AuNPs, red lines) compared to the unmodified electrode (SPE, black lines). This is due to the improvement in the charge transfer reaction of [Fe(CN)_6_]^−3/−4^ couple on the electrode as a consequence of the formation of nanoparticles with high electroactivity. Moreover, there was no significant increase in current for SPE/AuNPs above 100 s of electrodeposition, probably due to the electrode is saturated in that condition (Figure 6b,c). It is worth clarifying that the expected behavior of the electrode involves a reduction of the current in the immobilization of PEPTIR‑1.0 and detection of *E. coli* stages. This is because during each of these stages, the surface of the working electrode loses electroactivity. Thereby, the oxidation-reduction reactions of the [Fe(CN)_6_]^−3/−4^ couple will be restricted.

The difference between the current of the biosensor (SPE/AuNPs/PEP, blue lines) and the detection of the microorganism (SPE/AuNPs/PEP/EC, rose lines) is notably higher at potentials of electrodeposition of +0.05 V and −0.05 V at 100 s than at other potentials and times. Finally, the changes in the modification of the electrode to a reduction potential of −0.25 V are not marked.

**Figure 7:**
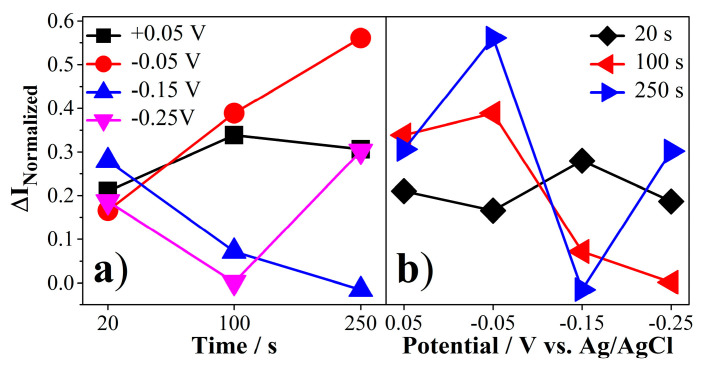
Two-factor factorial design results in the electrodeposition of AuNPs. (**a**) Normalized current vs. time of electrodeposition at +0.05 V (■), −0.05 V (●), −0.15 V (▲) and −0.25 V (▼). (**b**) Normalized current vs. applied potential at 20 s (▶), 100 s (◀) and 250 s (◆).

Normalized currents (ΔI_Normalized_) were obtained from voltammograms by using the maximum current value of SPE/AuNPs/PEP and the maximum current value of SPE/AuNPs/PEP/EC. The results obtained are shown in Table S1 (see supplementary information). The values in Table S1 were graphed to evaluate any simultaneous interaction between de potential and time as AuNPs electrodeposition variables on the biosensor response and the results are shown in Figure 7.

The results obtained for these variables show that they have an explicit influence on the response of the biosensor, i.e., the potential and time in chronoamperometry are independent variables but present an interaction which indicated that they are significantly influenced by each other. Moreover, a favorable trend was observed over time at a reduction potential of −0.05 V (Figure 7a, red curve). However, the tendency of the normalized current at 250 s is to decrease for all other potentials (Figure 7b, blue curve). Considering the above, it was established that a potential of −0.05 V and a time of 100 s are optimal to carry out the deposition of AuNPs by electrochemical reduction in this biosensor.

#### 2.3.2. Immobilization of PEPTIR‑1.0 on AuNPs‑Modified Screen‑Printed Electrodes

**Figure 8:**
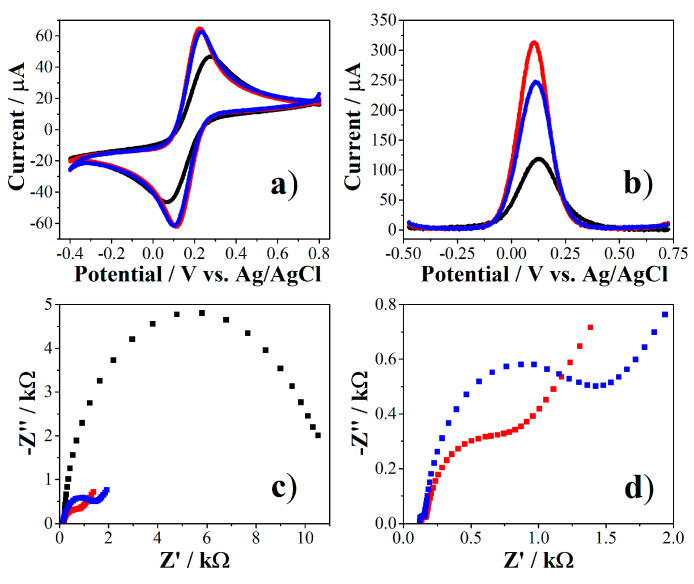
Electrochemical characterization of the screen‑printed electrodes modified with AuNPs and PEPTIR‑1.0 by CV (**a**), SWV (**b**) and EIS (**c**) during the construction of the biosensor. In all cases the black, red and blue lines or dots correspond to SPE, SPE/AuNPs and SPE/AuNPs/PEP, respectively. EIS results for SPE/AuNPs and SPE/AuNPs/PEP are presented expanded in (**d**) for clarity. 10 mM of [Fe(CN)_6_]^−3/−4^ in 0.1 M of KCl aqueous solution for CV and SWV. 2.0 mM of [Fe(CN)_6_]^−3/−4^ for EIS.

The PEPTIR‑1.0 peptide (sequence QKVNIDELGNAIPSGVLKDD) has a cysteine included in the *N*-terminal region of the chain. Thus, the immobilization of PEPTIR‑1.0 on the surface of AuNPs‑modified screen‑printed was performed by self‑assembly of the former through the thiol group on the surface of the AuNPs. The changes generated in the electrochemical properties of the working electrode (WE) were evaluated by cyclic voltammetry (CV), square wave voltammetry (SWV) and electrochemical impedance spectroscopy (EIS) by using [Fe(CN)_6_]^−3/−4^ couple as redox indicator and the results are shown in Figure 8.

As mentioned in the previous section, electrodeposition of AuNPs improves electrical transport properties of the surface of electrode. This was evidenced by CV and SWV as an increase in the current together with a decrease in the difference of the oxidation-reduction potentials of the [Fe(CN)_6_]^−3/−4^ couple in CV results (red curves in Figure 8a,b). Meanwhile, the Nyquist diagram in Figure 8c show that the unmodified electrode (SPE, black dots) exhibits the shape of a wide semicircle, indicating that the electrochemical process was dominated by electron transfer and the intrinsic resistance of the electrode is high. Then, the deposition of AuNPs modified the electrical behavior and a smaller semicircle is formed (red dots), indicating a decrease in the resistance of the electrode.

It was observed that the recognition molecule has the ability to modify the electrochemical properties of the surface of the WE as evidenced in the decrease of maximum of current in SWV (blue curve in Figure 8b). Moreover, the Nyquist diagram in Figure 8d (blue dots) reveals that the dative gold-sulphur (Au-S) bond disturbs the existing double layer at the electrode/electrolyte interface, generating a change in capacitance and increasing the electron transfer resistance [32,33]. These signal variations allow us to infer that the PEPTIR‑1.0 was able to change the diffusion phenomena of the ions present between the electrolyte solution and the electrode, which is associated with the successful immobilization of the molecule on the WE [34].

### 2.4. Evaluation of the Biosensor towards Detection of E. coli

#### 2.4.1. Evaluation of the Linear Response Range of the Biosensor

**Figure 9:**
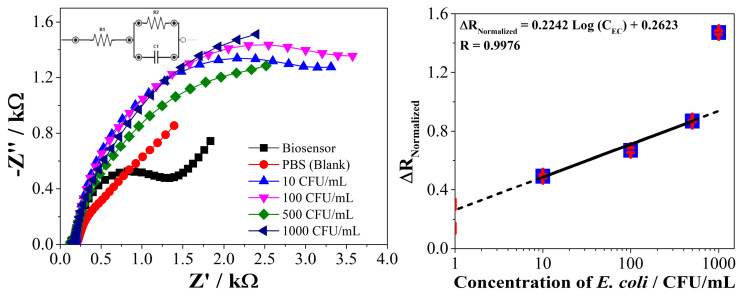
Results of electrochemical detection of *E. coli* (0 to 1000 CFU/mL) by using the screen‑printed electrodes modified with AuNPs and PEPTIR‑1.0 as recognition molecule. (**Left**): Nyquist diagram of the detection of 0 (blank, PBS), 10, 100, 500 and 1000 CFU/mL of *E. coli* (insert: Randles circuit used for fitting data). (**Right**): Correlation of normalized charge transfer resistance values (ΔR_Normalized_) with the concentrations of *E. coli*. 2.0 mM of [Fe(CN)_6_]^−3/−4^ in 0.1 M of KCl aqueous solution.

The biosensor obtained was evaluated in the detection of different concentrations (0, 10, 100, 500 and 1000 CFU/mL) of *E. coli*. The electrochemical response was monitored by electrochemical impedance spectroscopy (EIS) in the presence of [Fe(CN)_6_]^−3/−4^ couple as redox indicator. (Figure 9, left) shows the Nyquist diagram obtained from the EIS. These results were fitted to a Randles circuit (insert in Figure 9, left) to determine the charge transfer resistance (R_ct_) and capacitance values in each measurement [35,36]. Thus, it was possible to calculate the normalized charge transfer resistance values (ΔR_Normalized_) that were correlated with the concentration of the bacteria on a logarithmic scale (Figure 9, right).

The results show that the blank signal (PBS, red dots in Figure 9, left) modifies the biosensor response, probably due to the interaction of the ions in the buffer solution with the electrode surface. However, the bacteria considerably modify the electrical transport properties of the biosensor from the lowest concentration used (10 CFU/mL, blue triangles in Figure 9, left), compared to the blank. This is attributed to the formation of a complex between the PEPTIR‑1.0 and *E. coli* through of the Intimin membrane protein, which causes differences in the dielectric or conductivity properties and changing the rate of charge transfer [22]. Finally, it is important to highlight that the electrochemical properties of the negative control of the biosensor (SPE modified with AuNPs but without PEPTIR‑1.0) are not modified with the change in the concentration of *E. coli* (see supplementary information for details).

The normalized charge transfer resistance values change linearly with the concentrations of bacteria in a logarithmic scale. The basis from the electrochemical approach is that the interaction between the target bacteria and the PEPTIR‑1.0 block the charge transfer of the redox couple from the solution to the surface of the electrode. Nevertheless, the linear behavior is lost at concentrations of *E. coli* above 500 CFU/mL. It can be inferred that the biosensor is saturated with bacteria under the latter condition. It is important to clarify that it is necessary to first evaluate a broad range of concentrations of the analyte of interest before validating an instrument. In this sense, the results show a linear working range for the biosensor based on PEPTIR‑1.0 of around 0 to 500 CFU/mL.

It was possible to calculate the approximate limit of detection and quantification (LOD and LOQ) from the following equations:

(1) $$C_{m}=\frac{S_{m}-\bar{S_{bl}}}{m},$$

(2) $$S_{m}=\bar{S_{bl}}+kS_{bl},$$

where *C_m_* is the limit of detection or quantification, *S_m_* is the minimum distinguishable analytical signal, $\bar{S_{bl}}$ is the mean of the response of the blanks, *m* is the slope of the line, *S_bl_* is the standard deviation of the response of the blanks and *k* is a constant with 3 or 10 value for LOD or LOQ, respectively. Substitution of *S_m_* in *C_m_* results in

(3) $$C_{m}=\frac{kS_{bl}}{m}.$$

The calculated values of LOD and LOQ were 2 and 5 CFU/mL, respectively. These values show a highly response of the biosensor towards *E. coli*. These results contrast with that reported by Malvano, et al., who developed an impedimetric biosensor based on the antimicrobial activity of the peptide nisin for the detection of *Salmonella* spp. [22]. The authors report that the biosensor obtained is capable of selectively detecting *Salmonella spp* cells with a detection limit of 15 CFU/mL. On the other hand, Hoyos, et al. reported the design of an impedimetric sensor based on antimicrobial peptides for the early detection of periodontopathogenic bacteria [21]. The system is able to detect *S. sanguinis* in 1 h with LOD of 10 CFU/mL in KCl and 100 CFU/mL in artificial saliva. In the same context, Liu, et al. (2016) developed a biosensor based on synthetic peptides of modular design for the recognition, detection, and differentiation of pathogenic bacteria, including *E. coli* [37]. The system showed a detection limit of 100 CFU/mL of bacteria. According to the above, the system developed in the present study based on AuNPs and PEPTIR‑1.0 can be very promising for use in the detection of *E. coli* in aqueous systems. For this, it is necessary to perform a validation of the biosensor in the range of 0 to 500 CFU/mL.

#### 2.4.2. Evaluation of the Selectivity of the Biosensor

**Figure 10:**
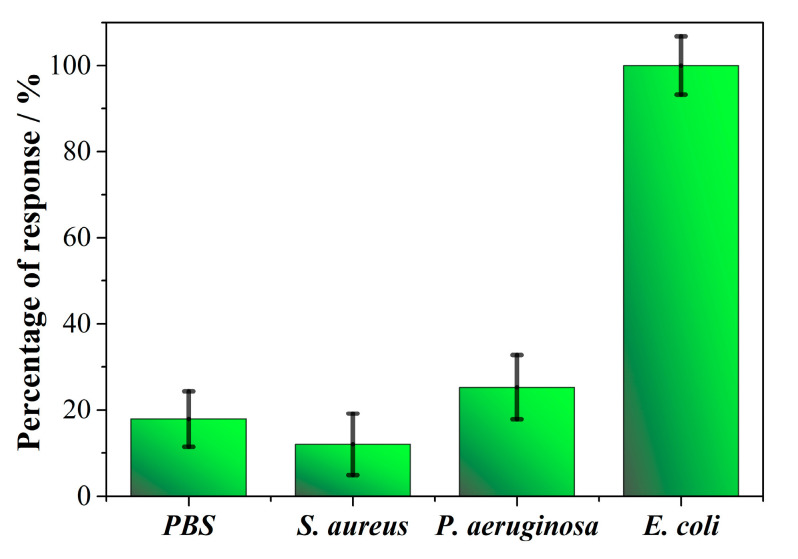
Selectivity results of the biosensor towards the detection of 50 CFU/mL of *E. coli*, *S. aureus* and *P. aeruginosa* bacteria.

The selectivity of the biosensor based on PEPTIR‑1.0 towards *E. coli* was evaluated in PBS solutions containing *S. aureus* and *P. aeruginosa* strains. For this, 50 CFU/mL of each microorganism were used and the changes in the impedimetric response of the biosensor were measured by EIS. The response of the biosensor in terms of percentage of selectivity towards each microorganism is shown in Figure 10.

The response of the biosensor to a model gram-positive bacterium such as *S. aureus* is comparable to blank. On the other hand, there is a slight response of the biosensor towards *P. aeruginosa* in comparison to blank. This can be due to the fact that this microorganism is gram-negative such as *E. coli*, considering that the biosensor recognizes whole‑cells. Despite the above, the signal belonging to the detection of *E. coli* (ΔR_Normalized_ = 1.27 ± 0.19, see Figure S4 in supplementary information) is higher and significantly different (*p*-value of 0.0295) than the other bacteria. This is attributed to the fact that the PEPTIR‑1.0 molecule was designed to interact with the Intimin membrane protein which is specific of *E. coli*. Other studies reported the development of an aptamer‑based impedimetric biosensor shows a LOD in the detection of *E. coli* O157:H7 of approximately 100 CFU/mL, with good selective response in the presence of *Salmonella typhimurium* and *Staphylococcus aureus* [37]. On the other hand, Yang, et al. developed an impedimetric biosensor without labeling based on a lectin-functionalized self-assembled mixed monolayer, showing a satisfactory selectivity to discriminate *E. coli* from other gram-positive bacteria [38].

Therefore, it is important to highlight the capabilities of the biosensor developed in the present study, with excellent LOD and LOQ values, but also the ability to discriminate the presence of *E. coli* from other bacteria. This make the PEPTIR‑1.0-based biosensor a promising system with high reliability for the detection of *E. coli* in aqueous matrices. Finally, it is necessary to carry out studies to evaluate nanostructures that allow to improve the electrical properties of the screen-printed electrode transducer.

## 3. Materials and Methods

### 3.1. Reagents, Materials and Instruments

All reagents were used as received without further purification. Potassium hexacyanoferrate (II) (K_4_[Fe(CN)_6_] × 3H_2_O, ≥98%, Merck, Darmstadt, Germany), potassium hexacyanoferrate (III) (K_3_[Fe(CN)_6_], ≥99%, Merck), gold (III) chloride hydrate (HAuCl_4_ × 3H_2_O, ≥99%, Sigma Aldrich, Saint Louis, MO, USA), potassium chloride (KCl ≥ 99%, Sigma Aldrich), LB-Agar (Merck).

The peptide PEPTIR‑1.0 (sequence QKVNIDELGNAIPSGVLKDD) was synthesized by Biomatik® (Wilmington, DE, USA) with a purity of >95%. A cysteine was included in the N-terminal region of the chain. The bacterial strains used were the references ATCC 43,895 *E. coli* O157:H7, ATCC 25,923 *S. aureus* and ATCC 27,853 *P. aeruginosa*.

Screen‑printed electrodes were acquired commercially (Palmsens) from the manufacturer Italsens, which consists of a working (7.07 mm^2^), an auxiliary carbon electrode and a silver/silver chloride (Ag/AgCl) reference electrode.

Electrochemical measurements and evaluation of the biosensor was performed on a potentiostat/galvanostat VersaSTAT 3 (Princeton Applied Research) controlled by Versastudio (v. 2.60.6.) software.

### 3.2. Selection of the TIR Protein Interaction Sequence and Its Modeling using Bioinformatics Tools

From the database RCSB Protein Data Bank the PDB 2ZQK file was obtained, which corresponds to the interaction model of the TIR and Intimin proteins of *E. coli* O157:H7 proposed by Ma, Y., Zou, Q. and Gao, GF [39]. The three-dimensional structures of the proteins were analyzed through the Chimera software to establish the domains or chains of interest and their possible areas of interaction [40]. Subsequently, through the LigPlot+ program [27], the amino acids that interact by hydrogen bonds and hydrophobic contacts between the Intimin and TIR proteins were selected. For the selected amino acid sequence, simulations were performed using the PEP-FOLD program [41], which is an online system that is based on the concept of the structural alphabet, a set of conformations of elemental prototypes capable of describing all the diversity of protein structures, allowing to perform the 3D reconstruction of peptides. Finally, among the five resulting models, the modeled structure that represented the smallest structural difference with the original A chain of the TIR protein in the 2ZQK model were selected, taking into account the RMSD (for its acronym “Root-mean-square deviation of atomic positions”), obtained from the structural alignment using the command: align name_String1, name_String2 in the PyMol program [42].

### 3.3. Selection of Ligands and Docking Molecular between the Peptide and the Intimin E Protein

From the 2ZQK model, the A chain was selected, which corresponds to the 3D structure of the Intimin protein. This chain is displayed together with the peptide model (*N*-chain) previously obtained with the PEP-FOLD program to carry out molecular docking simulations, which refers to the study of the interaction capacity between a ligand (peptide) and a receptor (Intimin protein). These simulations were carried out using Rosetta software, a program that allows, through its Refinement FlexPepDock and FlexPepDock ab-initio protocols, to create complex high-resolution models between peptides and proteins by iteratively optimizing the peptide skeleton and its rigid body orientation relative to the receptor protein [43].

### 3.4. Preparation and Characterization of the Biosensor

The electrodeposition of gold nanoparticles (AuNPs) on working electrode (WE) of the screen-printed electrodes (SPEs) was carried out by chronoamperometry (CA) through reducing of a 1.0 mM of HAuCl_4_ × H_2_O in 0.5 M of H_2_SO_4_ aqueous solution. Prior to AuNPs deposition, SPEs were previously cleaned with 0.5 M of H_2_SO_4_ and 0.1 M of KCl solutions by cyclic voltammetry (CV) by applying two cycles between +0.7 and −0.2 V and a scan rate of 0.05 V/s. Then, 100 μL of the gold precursor solution were placed on the SPEs and a reduction potential between +0.05 and −0.25 V during 20 to 500 s were applied. The selection of potentials was based on the results obtained by linear sweep voltammetry of the gold precursor solution. The influence of varying the potential and time in chronoamperometry on the response of biosensor were studied.

The peptide selected as a recognition molecule (PEPTIR‑1.0) has a cysteine included in the *N*‑terminal region of the chain to induce the formation of a stable S-Au bond with AuNPs. Thereby, the immobilization of the peptide was carried out by chemosorption. For this, 20 μL of a PEPTIR‑1.0 aqueous solution were placed only on the surface of WE and was left in incubation for 16 h at 25 °C [19,20]. After that, SPEs were rinsed with ultra‑pure water and 0.1 M KCl aqueous solution. The concentration of PEPTIR‑1.0 aqueous solution on the response of biosensor was studied.

Surface structural properties of working electrodes were studied by scanning electron microscopy (SEM) by using a Quanta Field Emission Gun (Model 650) microscope operated at 15.0 kV. The images were obtained in secondary electron mode.

**Figure 11:**
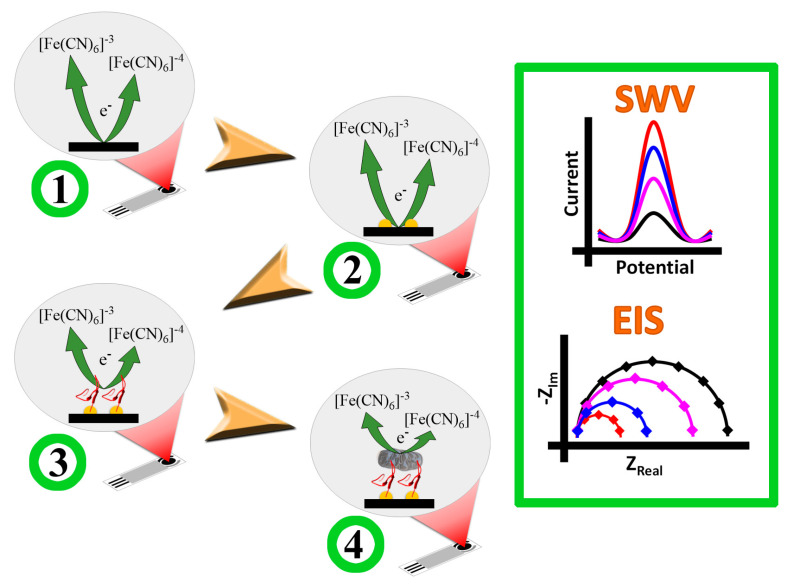
Diagram of the construction of the biosensor and the detection of *E. coli*. The electrochemical behavior of unmodified (SPE) the electrode in step 1 (**left**) is schematically represented by black lines in SWV and EIS curves (**right**). The expected response of the electrode is as follows: SPE/AuNPs (step 2 in left, red lines in SWV and EIS), SPE/AuNPs/PEP (step 3 in left, blue lines in SWV and EIS), and SPE/AuNPs/PEP/EC (step 4 in left, rose lines in SWV and EIS).

The electrochemical properties of the SPEs through each modification with AuNPs and PEPTIR‑1.0 were evaluated by cyclic voltammetry (CV), square wave voltammetry (SWV) and electrochemical impedance spectroscopy (EIS), using a potassium hexacyanoferrate (II)/(III) aqueous solution as redox probe and KCl (0.1 M) as supporting electrolyte (KCl is necessary for the proper functioning of the pseudo reference electrode). CV measurements were performed between +0.8 to −0.4 V leaving 10 s of stabilization or equilibrium at +0.8 V and a scan rate of 50 mV/s. SWV were performed between −0.4 to +0.8 V allowing 10 s of stabilization or equilibrium at −0.4 V, the amplitude or “Pulse Height” was 50 mV, the potential step (E_step_) was 5.0 mV and a frequency of 10 Hz. EIS measurements were performed between 50,000 to 1 Hz at a fixed potential of 10 mV RMS and +0.0 V vs. OCP. CV and SWV were performed using a 10 mM solution of redox probe while EIS measurements were performed using 2.0 mM of redox probe. Figure 11 (steps 1–3) shows the entire biosensor preparation scheme.

### 3.5. Detection of E. coli Cells Using the Prepared Electrochemical Biosensor

*Escherichia coli* O157:H7 (ATCC 43895) was kept under cryopreservation at −80 °C in Luria‑Bertani broth (LBB) with 15% of glycerol. For the reactivation of the microorganism, 50 µL of cryopreserved material was added in 5 mL of LBB and incubated at 35 ± 2 °C from 18 to 24 h before each assay, adjusting the concentration at 1 × 10^8^ CFU/mL in 10 mM phosphate buffer solution (PBS) at pH 7.4.

The evaluation of the biosensor on the detection of *E. coli* (EC) was performed as described below. 7 mL of a PBS solution (pH 7.4) with known concentrations of EC (10 to 1000 CFU/mL) were placed in an appropriate beaker and the biosensor was immersed. The system was incubated at 25 °C during 30 min and constant stirring of 150 rpm. After that, the biosensor was rinsed with ultra‑pure water prior to electrochemical measurements. The detection blank was the PBS solution without bacteria (0 CFU/mL). CV, SWV and EIS measurements were performed using the same conditions used for the characterization of the biosensor (Figure 11, step 4, see previous item).

The specific interaction of EC with the surface of WE limits the electron charge transfer between redox probe and the transductor. Therefore, current decreases in the case of CV and SWV and resistance to charge transfer increase in EIS [44]. On the other hand, to verify the ability of PEPTIR‑1.0 to show an interaction selectivity, solutions containing different bacteria were tested. In particular, the detection measures were performed in the presence of the strains: ATCC 43,895 *E. coli O157: H7*, ATCC 25,923 *S. aureus* and ATCC 27,853 *P. aeruginosa*, comparing the impedimetric results. The data analyzes to evaluate the EC detection were carried out from SWV and EIS measurements. The current normalized values (ΔI_Normalized_) and the resistance normalized values (ΔR_Normalized_) were calculated from SWV and EIS results, respectively, using the following equations:

(4) $$\Delta I_{Normalized}=\frac{I_{PEP}-I_{PEP+EC}}{I_{PEP}}$$

(5) $$\Delta R_{Normalized}=\frac{R_{PEP+EC}-R_{PEP}}{R_{PEP}}$$

where *I_PEP_* is the maximum current value of the biosensor (electrode modified with AuNPs and PEPTIR‑1.0), *I_PEP+EC_* is the maximum current value of the biosensor in the detection of bacteria, *R_PEP_* is the resistance to charge transfer of the biosensor and *R_PEP+EC_* is the resistance to charge transfer in the detection of bacteria. With these results, the current and resistance normalized values were correlated with the concentration of *E. coli* for establishing criteria such as the detection limit and linearity.

## 4. Conclusions

A new peptide was designed (named PEPTIR‑1.0) by bioinformatic tools based on the TIR protein, which is an *E. coli* receptor protein. PEPTIR‑1.0 was used as a recognition element in a biosensor based on AuNPs‑modified screen‑printed electrodes, allowing the sensible and specific detection of *E. coli* in an aqueous matrix.

It was possible to obtain a peptide sequence (QKVNIDELGNAIPSGVLKDD) from the TIR protein that interacts with the Intimin membrane protein through hydrophobic and electrostatic interactions. This sequence was shown to have a conserved three‑dimensional structure relative to the original structure in the protein.

On the other hand, it was evidenced that the structural characteristics of the AuNPs obtained by electrodeposition considerably affect the behavior of the biosensor. In addition, it was shown that the applied potential and the electrodeposition time of chronoamperometry are affected simultaneously. For this reason, these two variables must be evaluated simultaneously in each biosensor system.

The biosensor obtained based on PEPTIR‑1.0 exhibit a linear working range between 0 to 500 CFU/mL and limits of detection and quantification of 2 and 6 CFU/mL, respectively. Moreover, the statistically significant differences in the impedimetric responses of the biosensor in the presence of other microorganisms such as *S. aureus* and *P. aeruginosa*, highlight the possibility that this new biosensor can be used in the rapid, sensitive, and selective detection of *E. coli* in aqueous matrices.

## Figures and Tables

**Table 1:** RMSD values associated with the five models obtained with PEP-FOLD.

Model	RMSD
1	4834
2	7722
3	4252
4	8359
5	6011

**Table 2:** Interaction parameters between peptide models and the Intimin protein obtained with the FlexPepDock and Prodigy Haddock programs.

Model	ΔG (Kcal/mol) ^1^	Kd (mol/L) ^2^	Interactions	dG_Sep (Rosetta Energy)	dSASA_int (Å^2^)	Per_res_Energy (Rosetta Energy)
TIR (Original)	−11.50	3.7 × 10^−9^	62	−11.36	1174.01	+2.148
3.1	−10.9	9.6 × 10^−9^	52	−25.08	984.05	−1.873
3.2	−10.6	1.6 × 10^−8^	53	−25.19	981.98	−1.751
3.3	−10.8	1.3 × 10^−8^	47	−24.99	890.17	−1.845
3.4	−10.8	1.2 × 10^−8^	49	−22.52	998.71	−1.568
3.5	−10.5	2.1 × 10^−8^	54	−21.99	963.32	−1.745

^1^ Predicted value of the free energy of binding. ^2^ Predicted value of the dissociation constant (Kd) calculated from ΔG = RT ln (Kd), where R is the ideal gas constant (kcal K^−1^ mol^−1^) and T the temperature (K).

## Data Availability

Not applicable.

## Supplementary Materials

The following are available online, Table S1. Normalized current values obtained from SWV results for the potential and time used in electrodeposition of AuNPs; Figure S1. Results of SWV of the effect of the applied potential in chronoamperometry for AuNPs electrodeposition at a constant time of 20 s; Figure S2. Results of SWV of the effect of the applied potential in chronoamperometry for AuNPs electrodeposition at a constant time of 250 s.

## Sample Availability

Samples of the PEPTIR‑1.0 are available from the authors.
