# Supplementary material for: A Bioinspired Peptide in TIR Protein as Recognition Molecule on Electrochemical Biosensors for the Detection of E. coli O157:H7 in an Aqueous Matrix
Source: Molecules. 2021 Apr 28;26(9):2559. doi: 10.3390/molecules26092559 (PMC8124904; doi:10.3390/molecules26092559)
Supplement: Supplementary file 1 [file molecules-26-02559-s001.zip › molecules-1145482-supplementary.pdf]

Supplementary information

A bioinspired peptide in TIR protein as recognition molecule on electrochemical biosensors for the detection of E. coli O157:H7 in aqueous matrices

J.L. Ropero Vega [1,*], J.F. Redondo-Ortega [1,*], Y.J. Galvis-Curubo [1], P. Rondón-Villarreal [2], J.M. Flórez-Castillo [1]

[1] Universidad de Santander. Facultad de Ciencias Exactas, Naturales y Agropecuarias. Ciencias Básicas y Aplicadas para la Sostenibilidad-CIBAS. Calle 70 No. 55-210, C. P. 680003. Bucaramanga (Santander), Co-lombia; jose.ropero@udes.edu.co

[2] Universidad de Santander. Facultad de Ciencias de la Salud. MASIRA. Grupo de Investigación en Biología Molecular y Biotecnología-BIOMOL. Calle 70 No. 55-210, C. P. 680003. Bucaramanga (Santander), Colombia; diseno.molecular@udes.edu.co

* Correspondence: jose.ropero@udes.edu.co (J.L. Ropero Vega), jredondo@mail.udes.edu.co (J.H.F. Redon-do Ortega); Tel.: +57 7 6516500 Ext. 1665.

<u>Content</u>

1. **Normalized current values for the detection of *E. coli* (500 CFU/mL) obtained from SWV results for the different time and applied potential used in AuNPs electrodeposition.**

2. **Evaluation of the reduction potential for the electrodeposition of AuNPs on screen-printed electrodes (SPE, Italsens).**

3. **Negative control for the biosensor.**

4. **Selectivity of the biosensor towards the detection of *E. coli*.**

1. **Normalized current values for the detection of *E. coli* (500 CFU/mL) obtained from SWV results for the different time and applied potential used in AuNPs electrodeposition.**

Table S1. Normalized current values ($\Delta I_{Normalized}$) obtained from SWV results for the potential and time used in electrodeposition of AuNPs.

Time of electrodeposition	Potential of electrodeposition			
	**+0.05 V**	**-0.05 V**	**-0.15 V**	**-0.25 V**
20 s	0.210	0.166	0.280	0.187
100 s	0.339	0.389	0.072	0.002
250 s	0.306	0.561	-0.015	0.302

2. **Evaluation of the reduction potential for the electrodeposition of AuNPs on screen-printed electrodes (SPE, Italsens).**

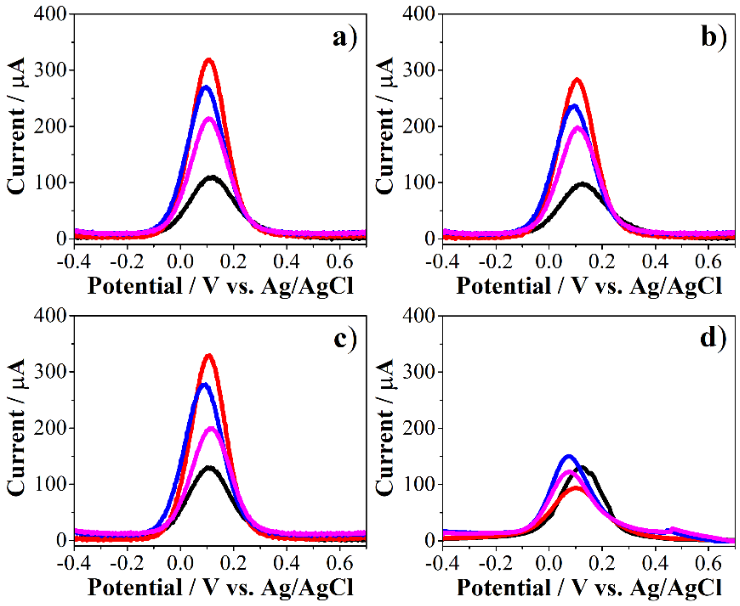

Figure S1. Results of SWV of the effect of the applied potential of +0.05 V (a), -0.05 V (b), -0.15 V (c) and -0.25 V (d) in chronoamperometry for AuNPs electrodeposition at a constant time of 20 s. The concentration of PEPTIR-1.0 and *E. coli* was 500 nM and 500 CFU/mL, respectively (concentrations used as reference). The curves correspond to SPE (black lines), SPE/AuNPs (red lines), SPE/AuNPs/PEP (blue lines) and SPE/AuNPs/PEP/EC (rose lines) in all cases. 10 mM $[Fe(CN)_6]^{-3}/[Fe(CN)_6]^{-4}$ in 0.1 M of KCl.

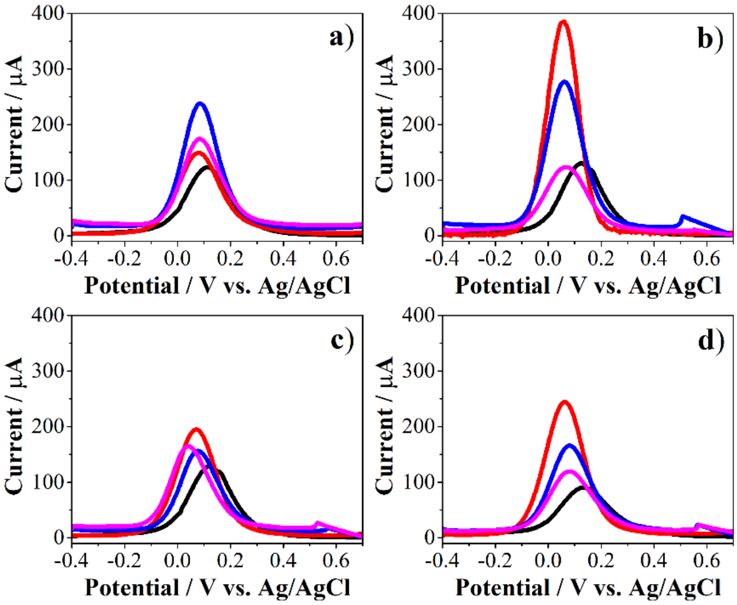

Figure S2. Results of SWV of the effect of the applied potential of +0.05 V (a), -0.05 V (b), -0.15 V (c) and -0.25 V (d) in chronoamperometry for AuNPs electrodeposition at a constant time of 250 s. The concentration of PEPTIR-1.0 and *E. coli* was 500 nM and 500 CFU/mL, respectively (concentrations used as reference). The curves correspond to SPE (black lines), SPE/AuNPs (red lines), SPE/AuNPs/PEP (blue lines) and SPE/AuNPs/PEP/EC (rose lines) in all cases. 10 mM $[Fe(CN)_6]^{-3}/[Fe(CN)_6]^{-4}$ in 0.1 M of KCl.

3. Negative control for the biosensor.

The negative control of the biosensor consists of a AuNPs-modified screen-printed electrode but without modification with PEPTIR-1.0. The results obtained are shown in Fig. S3.

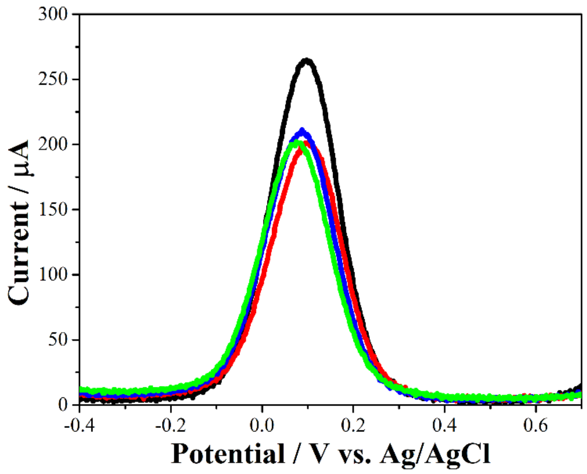

Figure S3. Results of SWV for the negative control of the biosensor. The curves correspond to SPE/AuNPs (black), SPE/AuNPs in blank of detection (PBS without *E. coli*, blue), SPE/AuNPs with 10 CFU/mL of *E. coli* (green) and SPE/AuNPs with 1000 CFU/mL of *E. coli* (red). Note that the PBS solution alone produces a change in the biosensor signal. Nevertheless, this signal is not affected by the presence of bacteria in the solution.

4. **Selectivity of the biosensor towards the detection of E. coli.**

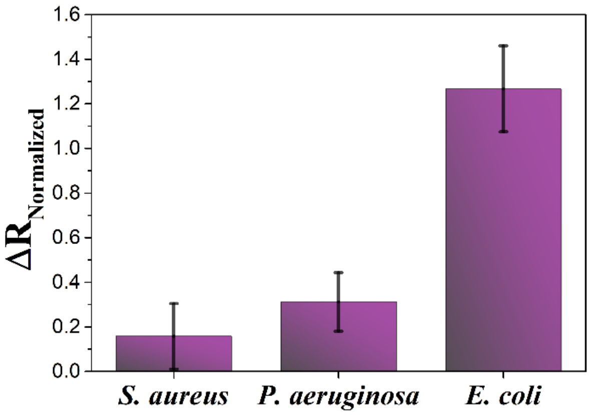

Figure S4. Normalized resistance values ($\Delta R_{Normalized}$) in the evaluation of the selectivity of the biosensor towards the detection of 50 CFU/mL of *E. coli*, *S. aureus* and *P. aeruginosa* bacteria.
